# Editorial: Epithelial-mesenchymal transition (EMT) as a therapeutic target in cancer

**DOI:** 10.3389/fonc.2023.1121416

**Published:** 2023-01-25

**Authors:** Weilong Zhong, Tao Sun

**Affiliations:** ^1^Department of Gastroenterology and Hepatology, General Hospital, Tianjin Institute of Digestive Diseases, Tianjin Key Laboratory of Digestive Diseases, Tianjin Medical University, Tianjin, China; ^2^State Key Laboratory of Medicinal Chemical Biology and College of Pharmacy, Nankai University, Tianjin, China

**Keywords:** epithelial-mesenchymal transition, metastasis, drug target, tumor therapy, Traditional Chinese medicine (TCM)

Tumor local invasion and metastases are the main factors leading to death of patients, and their occurrence depends on the malignant evolution of tumor cells (1). Epithelial–mesenchymal transition (EMT) is a major theory used to explain malignant progression. The phenomenon of EMT, first observed in the formation of embryonic gastrum and neural crest, is a precise regulatory mechanism of cell population differentiation. In cancer, EMT is closely associated with tumor metastasis but is also related to the maintenance of cancer stem cells (2). The occurrence of EMT requires specific biological signals, the generation of which being in part dependent on microenvironmental conditions. In particular, local tumor microenvironment stress induces the production of stress molecules, which in turn induces the expression of transcription factors prone to support EMT (3). The occurrence of EMT can enhance the invasiveness, anti-apoptotic ability, and drug resistance of tumor cells (4). Therefore, inhibition of EMT can be an important direction for the development of antitumor drugs.

In this research, Jiang et al. analyzed the latest research advancements in the contribution of exosomes to the regulation of the EMT process in tumor cells. Moreover, the author summarized the potential and challenges of using exosomes as a tool for cancer treatment.

Wang et al. focused on the role of TGF-β1 in autophagy and apoptosis in breast cancer. Their results showed that TGF-β1 promotes autophagy and inhibits the apoptosis of breast cancer cells by inhibiting the expression of TP63. This study provides a potential marker for the prognosis of breast cancer and a potential target for the treatment of breast cancer.

Zheng et al. identified multiple oncogenic genes (e.g., midkine [MDK]) associated with tumor metastasis. Their study found that IFN-γ treatment can induce the activation of the EMT process in a variety of cancer cell lines and even promote tumor metastasis. Furthermore, MDK is a common response target for IFN-γ in these cell lines. Ultimately, they suggested that targeted inhibition of MDK can eliminate IFN-γ-induced metastatic cancer from all sources. The combined use of MDK may expand the use of IFN-γ in cancer and improve the clinical efficacy of IFN-γ therapy.

Song et al. analyzed and reported the research trends and hotspots of glioma stem cells (GSCs) in the world in recent years through bibliometrics. The authors analyzed the most cited articles. Their results showed that “epithelial–mesenchymal transition” and “immunotherapy” have become new focus issues. Geographically, the United States is a leader in the field of GSC research. The focus of GSC research has gradually shifted from basic cell biology to clinical problem solving.

The field of cancer metabolism has evolved from a simple Warburg effect model to an understanding of the complexity of tumor metabolism, but some key issues still need to be addressed in the future. In the past few decades, the development and application of new technologies have not only revealed the heterogeneity and plasticity of tumors but also identified new metabolic pathways to sustain tumor growth. Yang et al. reported that ginseng nanoparticles inhibit EMT in lung cancer cells by inhibiting pentose phosphate pathway activity. We describe the typical characteristics of GDNP and the possible underlying mechanisms of GDNP’s antitumor activity. Our study showed that GDNPs induced the downregulation of thymidine phosphorylase (TP) expression, which led to the inhibition of the pentose phosphate pathway and lung cancer cell metastasis. As a new and promising anticancer drug, nanomaterials are worthy of further study.

Circulating tumor cells (CTCs) are tumor cells that shed from the primary site or metastasis and enter the bloodstream during tumor formation and progression. The formation of CTCs runs through the whole process of tumor development. The CTC test may represent an important new type of “liquid biopsy.” The number and type of CTCs are helpful in the differential diagnosis of early hepatic malignancies. Li Hua et al. systematically reviewed the clinical application of CTCs in hepatocellular carcinoma (HCC). The authors suggested that EMT plays a crucial role in distant metastasis. EMT also contributes to the powerful aggressiveness of CTCs. CTCs can also provide complete cell biology information, making CTCs one of the most promising targets for liquid biopsies. The clinical application of CTCs in HCC has a broad prospect.

Chen et al. reported that puerarin inhibits oxaliplatin (OXA)-induced EMT by targeting carbonic anhydrase (CA) XII. This study evaluated the anticancer effects of puerarin combined with OXA *in vitro* and *in vivo*. The author found that puerarin can reverse the resistance of platinum anticancer drugs and enhance the antitumor effect of OXA on breast cancer. Mechanistic studies suggested that CA XII is a potential target of puerarin. Puerarin has potential as an adjuvant chemotherapy drug and may be one of the “drug and food homologous” molecules in patients with breast cancer.

Zhang et al. investigated the expression of ZEB1 in three cases of hepatocellular sarcoma (HCS). Liu et al. found that during the malignant evolution of HCS, cancer cells acquire the mesenchymal phenotype and lose epithelial properties through the dynamic process of EMT. EMT-related transcription factor ZEB1 is highly expressed in sarcoma components. The high expression of ZEB1 in the HCS nucleus may be the key factor promoting EMT.

Cui et al. analyzed a novel EMT-related gene signature of predictive value for the survival outcomes in lung adenocarcinoma. They believe that the risk model based on E-signature is superior to the risk model reported in the literature. In patients with LUAD, E-signature risk scores are associated with stages T, N, M, and TNM. Their study explored an innovative EMT-based prognostic signature that may serve as a potential target for personalized and precision medicine.

Overall, EMT is an important cancer cell phenotype leading to tumor metastasis and drug resistance (5). Inhibitors of this cellular process can be used as chemotherapy or targeted therapeutic agents to provide clinical strategies for improving the efficacy of cancer therapy. Although many preclinical models have shown that EMT is involved in tumor metastasis, translating EMT into clinical application remains difficult. Targeting EMT is considered a new therapeutic direction to overcome cancer drug resistance. This topic includes some studies on the biological characteristics of EMT in tumor cells and its multilevel regulatory mechanisms. In addition, the relationship between EMT and cancer metastasis and the use of EMT as a potential therapeutic target were reported. In the personalized medicine of patients with cancer, EMT may serve as a useful therapeutic target to identify and intervene with disease-related EMT regulators, which is expected to promote the development of precision medicine and bring about technological changes in disease diagnosis and treatment.

## Author contributions

All authors listed have made a substantial, direct, and intellectual contribution to the work and approved it for publication.
